# Editorial: Social and affective domain in home language development and maintenance research

**DOI:** 10.3389/fpsyg.2024.1530140

**Published:** 2024-12-19

**Authors:** Anastassia Zabrodskaja, Sviatlana Karpava, Natalia Ringblom

**Affiliations:** ^1^Baltic Film, Media and Arts School, Tallinn University, Tallinn, Estonia; ^2^Department of English Studies, University of Cyprus, Nicosia, Cyprus; ^3^Department of Language Studies, Umeå University, Umeå, Sweden

**Keywords:** multilingualism, language maintenance, family language policies, bilingualism, language education, socialization processes, language transmission, cultural identity

Multilingualism leads to language contact, where individuals who speak different languages interact. This can result in either language maintenance, which ensures the transmission of a language across generations, or language shift, where a heritage language is abandoned in favor of a more dominant one. Language shift can occur within a single generation, often when parents consciously decide not to pass on a heritage language, or when children choose not to speak it. In such cases, the decision to raise children bilingually is typically a deliberate one, influenced by a variety of motivations.

In many cases, formal education systems prioritize proficiency in only one language, usually the dominant language of the region or country. However, language maintenance and transmission are more likely to succeed when the new generation recognizes the value of bilingualism. This recognition often arises when children see proficiency in multiple languages as offering social, economic, or cultural advantages. Active support from both parents and their wider social networks is crucial for sustaining bilingualism.

The aim of this Research Topic is to explore the key issues surrounding home language maintenance and development by bringing together scholars from various disciplines to address a range of interconnected Research Topics. These include multilingualism and its broader implications, family language policies and practices, and the role of digital literacies and digital practices in shaping language development. The research also examines socialization processes within bilingual families, and the impact of media on language use in bilingual households. The role of education in both home language development and bilingual education, as well as the unique challenges of special education in multilingual contexts, is explored. Central to the discussion is the perspectives of parents and teachers on the maintenance and development of home languages, contributing to a comprehensive understanding of how language is nurtured and transmitted across generations in diverse multilingual environments.

This research seeks to better understand the complex dynamics of multilingualism within the home and educational settings, highlighting the critical role of family, community, and policy in shaping language outcomes across generations.

The studies in this Research Topic underscore the complexities of language transmission, linguistic and cultural identity, and integration challenges. They explore various aspects of language dynamics, including language education and enjoyment, teacher agency and adaptation, and the impact of family language policies and practices. Additionally, the articles examine language proficiency and listening comprehension, offering instructive insights into how language skills develop in different socio-cultural contexts. Together, these studies provide a comprehensive examination of how language transmission and identity intersect with broader social, educational, and familial factors.

Sun presents a framework on the Harmonious Bilingual Experience, linking parents' bilingual perceptions, language use, and proficiency to children's bilingual skills and social-emotional wellbeing. It highlights parents' key role in fostering balanced bilingualism and positive development.

Purpuri et al. examine the “feeling different” experience of bicultural bilinguals during language switching, tied to cultural values and behavior. It can lead to exclusion but often enriches personal growth and societal contributions, offering insights into cultural identity amid immigration challenges.

Protassova and Yelenevskaya analyze how the war in Ukraine has changed language policies in Russian-speaking immigrant families. They show that many families with Ukrainian roots now prioritize Ukrainian to strengthen cultural ties, while Russian is viewed negatively. Some families, however, still prioritize Russian for educational and professional benefits.

Pagé and Noels study how childhood language policies in multilingual families affect language retention in emerging Canadian adults. They find that most participants, aged 17–29, aim to retain their home language and are open to adding other languages, providing insights into effective heritage language retention across generations.

Ergün and Demirdaǧ explore how positive language education boosts foreign language enjoyment (FLE) via subjective wellbeing (SWB). Interventions improved classroom atmosphere and self-awareness. Results show SWB significantly predicts FLE, highlighting the role of positivity in language learning.

Szczepaniak-Kozak and Wa̧sikiewicz-Firlej investigate teacher agency in Polish schools after the 2022 Ukrainian refugee influx, highlighting teachers' swift adaptation to new linguistic and cultural diversity through collaboration and training, despite limited resources.

Nenonen scrutinizes positive attitudes toward multilingualism and the influence of social factors on language practices in a multilingual Russian-Italian family in Finland. The family uses an “one person-one language” strategy, with each parent speaking a different language to the child.

Schwartz and Ragnarsdóttir present a model for home-preschool continuity in linguistically and culturally diverse settings. They integrate responsive teaching, family language policies, and parental involvement, based on Bronfenbrenner's ecological model, Epstein's parental involvement model, and teacher-parent agency. The suggested model aims to support children's linguistic security through collaboration between parents and teachers, offering a framework for research and practical solutions in multilingual preschool settings.

Gacs et al. examine listening comprehension in German-Russian bilinguals aged 13–19, focusing on Russian as the home language. They explore how language proficiency, family input, and media exposure affect listening skills at various levels (phoneme, word, sentence, and text), finding differences in comprehension across these levels and highlighting the role of linguistic background and language input in shaping listening abilities.

This Research Topic provides valuable insights into the relationship between family language policies, bilingualism, and multilingual practices. The studies highlight the importance of supportive environments in both home and educational settings, showing how parents, educators, and communities play key roles in maintaining multilingualism. Together, the contributions emphasize the need for a comprehensive approach to language development that considers social, emotional, and cultural factors to ensure the sustainability of linguistic diversity across generations.

The practical implications are broad. It can inform language policies that support bilingual education and home language preservation. The findings also offer guidance for training parents and teachers to better support bilingual development. Targeted services for multilingual families can help address language maintenance and integration challenges. In education, the research can shape curricula that promote bilingualism and heritage language retention. Finally, it highlights the role of community networks in supporting language maintenance and fostering intercultural understanding. Future research could explore the long-term impacts of bilingual upbringing on cognitive, identity, and socio-economic development across different cultural contexts.

